# Enhanced resolution profiling in twins reveals differential methylation signatures of type 2 diabetes with links to its complications

**DOI:** 10.1016/j.ebiom.2024.105096

**Published:** 2024-04-04

**Authors:** Colette Christiansen, Louis Potier, Tiphaine C. Martin, Sergio Villicaña, Juan E. Castillo-Fernandez, Massimo Mangino, Cristina Menni, Pei-Chien Tsai, Purdey J. Campbell, Shelby Mullin, Juan R. Ordoñana, Olga Monteagudo, Perminder S. Sachdev, Karen A. Mather, Julian N. Trollor, Kirsi H. Pietilainen, Miina Ollikainen, Christine Dalgård, Kirsten Kyvik, Kaare Christensen, Jenny van Dongen, Gonneke Willemsen, Dorret I. Boomsma, Patrik K.E. Magnusson, Nancy L. Pedersen, Scott G. Wilson, Elin Grundberg, Tim D. Spector, Jordana T. Bell

**Affiliations:** a King's College London, UK; b APHP, Paris Cité University, INSERM, Paris, France; c Icahn School of Medicine at Mount Sinai, USA; d Department of Biomedical Sciences, Chang Gung University, Taoyuan City, Taiwan; e Molecular Infectious Disease Research Center, Chang Gung Memorial Hospital, Taoyuan City, Taiwan; f Department of Endocrinology & Diabetes, Sir Charles Gairdner Hospital, Nedlands, WA, Australia; g School of Biomedical Sciences, University of Western Australia, Crawley, WA, 6009, Australia; h University of Murcia, Spain; i University of New South Wales, Australia; j Obesity Research Unit, Research Program for Clinical and Molecular Metabolism, Faculty of Medicine, University of Helsinki, Finland; k Minerva Foundation Institute for Medical Research, Helsinki, Finland; l University of Southern Denmark, Denmark; m Department of Biological Psychology, Vrije Universiteit Amsterdam, the Netherlands; n Karolinska Institute, Sweden; o Children's Mercy Kansas City, USA; p The Open University, Milton Keynes, UK; q HealthyWeightHub, Abdominal Center, Helsinki University Hospital and University of Helsinki, Finland; r Institute for Molecular Medicine Finland, FIMM, HiLIFE, University of Helsinki, Finland

**Keywords:** DNA methylation, Type 2 diabetes, Genetics, Twins

## Abstract

**Background:**

Type 2 diabetes (T2D) susceptibility is influenced by genetic and environmental factors. Previous findings suggest DNA methylation as a potential mechanism in T2D pathogenesis and progression.

**Methods:**

We profiled DNA methylation in 248 blood samples from participants of European ancestry from 7 twin cohorts using a methylation sequencing platform targeting regulatory genomic regions encompassing 2,048,698 CpG sites.

**Findings:**

We find and replicate 3 previously unreported T2D differentially methylated CpG positions (T2D-DMPs) at FDR 5% in *RGL3, NGB* and *OTX2,* and 20 signals at FDR 25%, of which 14 replicated. Integrating genetic variation and T2D-discordant monozygotic twin analyses, we identify both genetic-based and genetic-independent T2D-DMPs. The signals annotate to genes with established GWAS and EWAS links to T2D and its complications, including blood pressure (*RGL3*) and eye disease (*OTX2*).

**Interpretation:**

The results help to improve our understanding of T2D disease pathogenesis and progression and may provide biomarkers for its complications.

**Funding:**

Funding acknowledgements for each cohort can be found in the Supplementary Note.

**Research in context**

**Evidence before this study**

Type 2 Diabetes is the most common metabolic disease with rapidly rising prevalence worldwide. DNA methylation has been proposed as a mechanism to mediate genetic and environmental risk factors in development and progression of T2D. Multiple DNA methylation alterations have been identified in individuals with T2D, but efforts to date have been predominantly based on assays that only explore <2% of the human methylome.

**Added value of this study**

This study explored the blood methylome in T2D targeting regulatory and functional genomic regions. The approach allowed for characterisation of genomic regions that display highly variable methylation signatures, and a subset of variable signals differentiated individuals with T2D. The study was able to assess whether the T2D differential methylation signatures had a genetic or non-genetic basis in genetically identical twins discordant for T2D. The peak T2D differential methylation signals targeted genes with links to clinical complications in T2D, and follow-up suggested that some methylation signals are altered specifically in individuals with T2D who develop complications.

**Implications of all the available evidence**

Distinct alterations to the human methylome occur with T2D. A subset of effects have a genetic basis or occur prior to T2D, suggesting a role for DNA methylation in T2D development. This study identifies evidence for a relationship between T2D methylation signals and development of T2D complications, which implicates DNA methylation in T2D progression. The results suggest that DNA methylation levels may be useful markers for risk of developing T2D complications.

## Introduction

Type 2 diabetes (T2D) prevalence is rising globally from an estimated 108 million cases in 1980^1^ to projections of around 700 million by 2045.^2^ It is a global health concern as chronic exposure to hyperglycemia induces clinical complications, including cardiovascular disease, visual impairment, lower limb amputation and renal disease. There is a clear genetic component to T2D with the most recent and largest multi-ancestry genome-wide association meta-analysis identifying 568 susceptibility loci, which account for around 50% of T2D heritability.^3^ In the EURODISCOTWIN consortium, which included nearly 70.000 twins, the heritability for T2D was estimated at 72% (95% confidence interval 61–78%).^4^ However, the rise in T2D also mirrors rising obesity rates globally, where the number of obese adults worldwide has tripled since 1975.^1^ This in part reflects changing lifestyles, with diet and lack of exercise as two of the environmental factors that contribute to disease pathogenesis. Epigenetic alterations have been proposed as a candidate mechanism to mediate genetic and environmental effects leading to T2D. Although multiple studies have linked DNA methylation changes to T2D, the role of epigenetics in T2D remains incompletely clear, including whether methylation is causal or secondary to disease onset, and how it relates to T2D complications.

Previous work has identified many differentially methylated CpG sites between individuals with T2D and those without, suggesting that DNA methylation could be a possible epigenetic mechanism underlying T2D pathogenesis. Multiple studies have been carried out in whole blood^5^, ^6^, ^7^, ^8^, ^9^ and in T2D relevant tissues including pancreatic islet cells,^10^, ^11^, ^12^, ^13^, ^14^, ^15^ liver^16^ and adipose^17^^,^^18^ tissue. The largest study to date was a longitudinal study in whole blood in 13,535 Indian Asian and 7066 European participants,^5^ contrasting DNA methylation profiles based on the Infinium HumanMethylation450 BeadChip array (450k) between individuals who developed T2D over 8 years (1608 Indian Asians and 306 Europeans) and those who did not. The findings replicated previous differential methylation T2D signals in *TCF7L2* and *KCNQ1,* and found previously unreported signals including in *TXNIP*, *ABCG1* and *SREBF1*, which have been subsequently replicated by other blood studies with more than 100 T2D cases.^6^, ^7^, ^8^, ^9^ More recent studies have also identified and replicated differential methylation in T2D at *CPT1A.**^9^* It is difficult to disentangle cause and effect, but the findings by Chambers et al.^5^ suggest that differential methylation at CpG sites in five regions (*TXNIP*, *SOCS3*, *PHOSPHO1*, *ABCG1*, *SREBF1*) plays a contributing role to future development of T2D over an 8 year follow up. Overall DNA methylation at *TXNIP*, *ABCG1*, *SREBF1*, *CPT1A*, *TCF7L2* and *KCNQ1* has been found to be associated with T2D in at least two independent whole-blood datasets. Except for signals in *TCF7L2* and *KCNQ*, two genes that also harbour genetic variants associated with T2D, the T2D blood-based DNA methylation signals appear to be tissue specific. T2D blood-based DNA methylation studies have not identified consistent association results. The only gene for which most studies report differential DNA methylation in T2D is *TXNIP*.

Most T2D DNA methylation studies to date have been carried out in unrelated individuals, but there have been a small number of studies in families and within monozygotic (MZ) twin pairs. MZ twins in a pair have almost identical genetic variation profiles, as they arise from a single fertilized egg, and are matched for age and sex, enabling analyses of the role of non-genetic factors on DNA methylation in T2D. These may include DNA methylation signals mediating T2D environmental risk factor effects, or signals that occur as a consequence of T2D. Four studies of MZ twin pairs who were discordant for T2D have examined the association between T2D and DNA methylation. These include two studies in whole blood, where the first was conducted in 17 MZ twin pairs discordant for T2D^19^ and identified a hypermethylated region in the promoter of *MALT1*, a gene with a key role in energy and insulin pathways. The second blood-based study of 11 MZ twin pairs discordant for T2D^20^ found differential methylation at cg18681426 in *ELOVL5*, a gene involved in the elongation of long-chain polyunsaturated fatty acids. Two further studies in adipose (14 twin pairs^17^), and in adipose and skeletal tissues (11 twin pairs^21^) found no significant results genome-wide after multiple testing correction. In addition to the studies in twins, a study of 39 families was carried out^22^^,^ which replicated the results from studies in unrelated samples of *TXNIP* and *ABCG1,* and also found differential methylation in *SAMD12*.

If the associated differential methylation signals occur as a consequence of T2D, they could unveil mechanisms through which T2D-related clinical complications develop. In contrast to the previous studies on T2D and DNA methylation, there has not been a consistent exploration of the effect of DNA methylation variation on T2D disease progression, especially in the development of T2D complications. In previous work when T2D cases were grouped into 4 subtypes (severe insulin-deficient diabetes, severe insulin-resistant diabetes, mild obesity-related diabetes, and mild age-related diabetes) reflecting different risks for developing T2D complications, the four T2D groups had different DNA methylation signatures in blood.^23^ For example, of 95 methylation sites found to discriminate between these four groups, 39 were annotated to genes previously linked to diabetes and related traits, including *TXNIP*.^23^ Increased expression of *TXNIP* has also been shown to cause oxidative stress, inflammation and apoptosis in retinal cells,^24^ and its inhibition blocks the early stages of diabetic retinopathy.^25^ Given these findings a more comprehensive study of DNA methylation changes and T2D complications may yield insights and provide biomarkers for developing complications in T2D progression.

Nearly all previous DNA methylation studies of T2D in blood, with the exception of Yuan et al.,^19^ have been carried out utilising the 450k array. However, this methylation array design does not take into account variation in DNA methylation levels across individuals and targets many CpGs that exhibit little inter-individual variability.^26^ In particular, the 450k array has a bias for promoter regions, which have lower variability and are less dynamic in response to exposures.^27^ Yuan et al.,^19^ explored whole blood DNA methylation profiles determined using methylated DNA immunoprecipitation sequencing (MeDIP-seq). This sequencing technology offers wider genome coverage, but lacks single base pair resolution and shows bias towards methylated regions. In contrast, a recently developed DNA methylation sequencing technology, methyl-C capture sequencing (MCC-seq), targets enhancers and functional areas of the genome leading to an enrichment in regulatory regions. Allum et al.^28^ demonstrate that MCC-seq is comparable in accuracy to the 450k array, but better targets disease-relevant regions. In addition to targeting disease-relevant regions, MCC-seq profiles 3.7 million sites, nearly an 8-fold increase on the 450k array. Allum et al.^29^ have also utilised the power of MCC-seq profiling to identify differential methylation in functional regions relating to cardiometabolic traits using circulating plasma lipid levels as proxies for cardiometabolic health.

Here we utilise MCC-seq to profile DNA methylation in whole blood samples from European ancestry participants from 7 international twin cohorts,^4^ including 113 T2D cases and 135 controls, of which a subset were 74 MZ twin pairs discordant for T2D. The aim was to determine associations between DNA methylation genome-wide and retrospective T2D status (T2D-DMPs). We explored the genetic drivers of T2D, and inclusion of 74 T2D-discordant MZ pairs enabled us to test for non-genetic drivers of T2D. T2D-DMP replication was pursued in an independent dataset of 573 individuals, including 33 T2D cases. T2D-DMPs were also validated using blood 450k profiles in 49 cases and 681 controls for association with T2D, and in 978 individuals for association with fasting blood glucose also profiled in the 450k array. T2D-DMPs were further investigated for their functional relevance by exploring their association with blood metabolomic profiles, and for clinical relevance with respect to the development of T2D-related complications. The results of this study have potential to improve our understanding of the molecular changes mediating genetic and environmental risk effects for T2D, and their interactions, and potentially act as biomarkers for T2D complications.

## Methods

### Study participants

The primary analysis in the study included 248 participants (135 controls and 113 T2D) from 7 international twin study cohorts including the Danish Twin Registry (DTR), The Finnish Twin Cohort Study (FTCS), Murcia Twin Registry (MTR), Netherlands Twin Register (NTR), The Older Australian Twins Study (OATS), The Swedish Twin Registry (STR), and TwinsUK (Table 1, Supplementary Note). The 248 individuals included in the main study were of European ancestry with the exception of two individuals of Indonesian ancestry in the NTR cohort. Overall, 85% of the sample consisted of monozygotic (MZ) twin pairs, including 74 MZ twin pairs discordant for T2D. The sex proportion varied across cohort samples, ranging from 18% to 100% female, and overall 61% of participants were female. Participants were predominantly sampled in mid-life or at an older age, from the youngest cohort samples in DTR (mean age 55 ± 7) to the oldest cohort samples from OATS (mean age 69 ± 4) and STR (mean age 70 ± 6). Whole blood DNA samples from the 248 participants were profiled for DNA methylation using MCC-seq as described below.

**Table 1:** Discovery sample participant characteristics.

Cohorts	n	Controls	Control BMI^b^ (kg.m^b^)	Control Age^a^	Control FBG	T2D	T2D BMI^b^ (kg.m^b^)	T2D Age^a^	T2D FBG
TwinsUK	93	48	27.5 ± 5.0	63.5 ± 13.0	4.9 ± 0.5	45	29.8 ± 6.0	60.9 ± 12.8	7.6 ± 4.3
Older Australian Twins Study	11	6	25.6 ± 2.7	69.4 ± 4.2	4.8 ± 1.0	5	30.5 ± 5.2	68.8 ± 4.1	6.7 ± 1.3
Finnish Twin Cohort Study	20	10	28.1 ± 4.8	60.4 ± 11	5.6 ± 0.4	10	33.9 ± 5.6	60.4 ± 11	7.6 ± 3.3
Netherlands Twin Register	46	33	26.2 ± 4.6	50.9 ± 15	5.3 ± 0.5	13	27.2 ± 3.8	59.2 ± 19	9.2 ± 2.8
Murcia Twin Registry	9	5	32.4 ± 2.6	61.1 ± 5.1	n/a	4	31 ± 5.4	62.1 ± 5.3	7.3 ± 1.4
Danish Twin Registry	22	11	28.1 ± 1.9	55.6 ± 6.7	5.9 ± 0.5	11	32 ± 5.7	55.7 ± 6.7	7.4 ± 1.0
Swedish Twin Registry	47	23	25.1 ± 3.9	69.7 ± 6	5.3 ± 0.3	24	28.8 ± 4.3	69.8 ± 5.8	7.6 ± 1.4
Total	**248**	**135**	**27** ± **4.5**	**60.7** ± **13.3**		**113**	**30** ± **5.4**	**62.5** ± **12.1**	

a No significant difference in age overall P = 0.23.

b Significant overall difference in BMI P < 0.001.

The validation dataset for the study included 978 individuals from the TwinsUK cohort (mean age 57.8 ± 10.7, mean BMI 26.7 kg/m2 ± 4.7) whose whole blood DNA methylomes were profiled using an alternate method, the 450k array. Of these, 730 participants were analysed as a case–control study of T2D (49 T2D cases and 681 controls), and all 978 individuals were included in the DNA methylation association analysis with fasting blood glucose. Altogether 44 of these participants (19 T2D cases and 25 controls) overlapped with the discovery participants in the primary analysis from the TwinsUK MCC-seq sample.

The replication dataset consisted of 573 TwinsUK individuals with whole blood MCC-seq profiles, of whom 33 had T2D at the time of blood collection (age 54.2 ± 13.4, BMI 25.7 kg/m2 ± 4.1). These individuals were predominantly female (93%) and the majority were MZ twins. They were free from major diseases at the time of sampling and metabolic health phenotypes in this sample were representative of the population. There was no overlap between the discovery and replication samples.

All study participants provided informed consent. Ethical approval was granted for all the cohort samples. Further details and a description of each cohort are provided in Supplementary Note 1.

### DNA methylation profiles

#### Methyl C capture sequencing profiles

Methylation was profiled using Methyl C Capture Sequencing (MCC-seq), a targeted bisulfite sequencing epigenome profiling approach, which has been previously described.^28^ In this study a capture panel designed to target functional regions in whole blood was used. This panel has been used in three other studies to date.^29^, ^30^, ^31^ Specifically, regions incorporated in the panel included 1) low and unmethylated regions (LMR/UMR) identified from merged whole genome bisulfite sequencing (WGBS) data from 30 whole blood samples derived from the TwinsUK cohort,^32^ 2) Immune/circulating leukocyte-specific regulatory regions identified by ChIP-Seq or DHS mapping and 3) Illumina 450k array CpG sites. In all, the panel captures 4,861,805 CpGs *via* 607,984 targeted regions. All targeted regions were merged, and a panel was generated by Roche NimbleGen.

The protocol for using the panel has been described previously.^28^ Briefly, following library preparation, bisulfite conversion and amplification of the MCC-seq library, targeted DNA fragments are captured, amplified again, and sequenced. Reads are then aligned to a bisulfite-converted hg19/GRCh37 reference genome, with the removal of poor-quality reads and mismatches. CpGs were also removed if not covered by at least five reads and at least two reads per strand. Further, CpGs were selected for less than 20% difference in methylation between strands. The methylation value was determined as total (forward and reverse) non-converted C over total (forward and reverse) reads. Sites were only included in the downstream analysis where missing values were limited to less than 20% of the individuals. Overall, this included 2,048,698 sites. Further details are provided in the Supplementary Note.

For many of the sequenced sites there was little variability between individuals. The standard deviation for the DNA methylation variability between all sites had a median of 2% (lower quartile 1.4% and upper quartile 4.2%). To ensure the analysis focused on sites which were variable between individuals, and as such more likely to be related to disease pathogenesis, we restricted our analysis to the top quartile of most variable CpG sites. Therefore, the downstream analyses focused on the 512,175 most variable CpG sites.

#### 450k DNA methylation profiles

DNA extraction in the whole blood TwinsUK validation samples has been described previously.^33^ DNA methylation profiling of these samples using the Ilumina HumanMethylation 450kBeadChip array (450k array) has also been previously described.^33^ Briefly DNA methylation levels used here are Illumina beta-values, defined as the ratio of the methylated probe intensity over the sum of methylated and unmethylated probe intensities plus 100.^34^ Methylation beta-values range between 0 at unmethylated CpG-sites, and 1 at fully methylated CpG sites. Methylation 450k data were processed, and quality control assessment was carried out using Enmix.^35^ Exclusions were determined using Minfi^36^ with samples with median methylated and unmethylated signals below 10.5 excluded. In addition, cross-reactive probes and probes containing >2 alignment mismatches were excluded. Altogether, 438,594 450k probes were included in the downstream analysis.

#### MeDIP-seq methylation profiles

DNA methylation in whole blood was profiled using methylated DNA immunoprecipitation sequencing (MeDIP-seq) in the TwinsUK cohort, as previously described.^37^^,^^38^ TwinsUK participants included the current MCC-seq study datasets were excluded from the TwinsUK MeDIP-seq dataset, resulting to a final independent TwinsUK MeDIP-seq dataset consisting of 116 T2D cases and 3318 controls. As previously described,^38^ sonication was used to fragment DNA, following which libraries were prepared using Illumina's DNA Sample Prep kit for single-end sequencing. Immunoprecipitation was then carried out using anti-5mC antibody (Diagenode) and qPCR used for validation. Captured DNA underwent purification and amplification, following which 200-500bp fragments were selected. Sequencing was then carried out using the Illumina Platform and aligned using BWA.^39^ Methylation levels were quantified using MEDIPS v1.0.^40^ After processing, MeDIPseq data was quantified in bins of 500bps with a 250bp overlap.

### Type 2 diabetes status and fasting blood glucose

Type 2 Diabetes (T2D) status was determined for all participants in the study, using a combination of approaches. We determined self-reported T2D status from questionnaire data, as well as based on circulating fasting glucose measurement of at least 7 mmol/L, or use of blood glucose lowering medication. T2D status information was obtained at the same clinical visit during which the blood sample for DNA methylation profiling was collected, with the exception of the OATS samples. In the OATS dataset if T2D status is based on self-reported status, then this was information was obtained within 3 months of the blood sample collection date. Full details of T2D status determination for each cohort are provided in the Supplementary Note.

### Peripheral blood cell proportions

Blood cell type proportions were estimated for monocytes, granulocytes, Natural Killer (NK) cells, CD8, CD4 and plasmablasts using the approach proposed by Houseman et al.^41^ The R package “FlowSorted.Blood.450k”^42^ was used to estimate blood cell type proportions based on the subset of 450k signals included in the MCC-seq panel. As expected, the estimated cell type proportions showed high levels of correlation and therefore not all estimated cell types were included in the model. The final model of analysis included covariates for granulocytes, NK cells and CD4 T cells, due to these cell types showing the lowest correlations between them. All other cell types were correlated (r > 0.7) with one of these variables.

### Epigenome-wide association analysis

An epigenome-wide association study (EWAS) was carried out to determine associations between whole blood DNA methylation variation and T2D case control status. DNA methylation values for each CpG-site were normalised to N (0,1) prior to fitting linear models. For the case–control analysis, a mixed effect linear model was fitted (using lme4 and LmerTest in R). Methylation at the CpG site was the response variable with T2D status as the predictor. Covariates included age, smoking status, sex, blood cell composition as fixed effects and family and zygosity as separate random effects. Methylation effect sizes were calculated using the same linear models, but without normalizing DNA methylation levels to N (0,1) prior to data analysis. A sensitivity analysis was carried out including BMI as an additional covariate. Multiple testing adjustment was carried out using Benjamini and Hochberg False Discovery Rate (FDR) thresholds of FDR 5%, and a more relaxed threshold of FDR 25%.

This mixed effects linear model was also applied in the replication analysis in testing the association of MCC-seq variation at each candidate CpG site with T2D status, and similarly in the validation analyses assessing the association of 450k methylation profiles at candidate sites and metabolic phenotypes.

We explored the genes that the T2D-DMPs arising from the case–control analysis mapped to using gene set enrichment analysis (GSEA),^43^ as previously described.^44^ We also compared the 16 T2D-DMP genes to nine major collections of gene sets within the Human Molecular Signatures Database (MSigDB (http://www.gsea-msigdb.org/gsea/msigdb/index.jsp). We report enrichment results at a significance value of FDR 5%.

### Genetic independent (discordant MZ twin) analysis

A subset of the data was used to carry out a T2D-discordant MZ twin pair analysis, focusing on MZ twin pairs alone. Ascertainment into the study prioritized inclusion of 74 pairs of MZ twins who were discordant for T2D in the main MCC-seq dataset. T2D-discordant MZ twin pair analysis was performed for differential methylation at the T2D-DMPs identified by the case control analysis at a FDR 25% threshold. Statistical significance was determined using a one-sample parametric t-test for paired samples. This tested whether the mean methylation difference within each twin pair of the covariate-adjusted methylation data was significantly different from zero. Covariate-adjusted methylation data were generated using a linear mixed effects model including methylation as the response variable, and covariates from the case control analysis (age, sex, smoking status, blood cell composition) as fixed effect variables and family and zygosity as random effects.

### Validation analysis

The MCC-seq validation analyses using 450k data targeted the FDR 25% results from the MCC-seq T2D case–control analysis. We used 450k DNA methylation levels at the target CpG site, or at a 450k CpG-site nearest to the target, and assessed their association with T2D and fasting blood glucose. In comparing MCC-seq to 450k data we used overlapping CpG sites where possible. Only 5 of the 20 T2D-DMPs were profiled on the 450k array, with the majority being unique to the MCC-seq platform. In instances where there was no matching site available, we selected the nearest 450k CpG site for validation testing, where the furthest distance between nearest 450k CpG to target CpG was 2.7 kB. To assess significance accounting for multiple testing, a Bonferroni threshold of P = 0.0025 (P = 0.05/20) was applied. Validation analyses were also carried out using the independent MeDIP-seq dataset from TwinsUK. For MeDIPseq validation we analysed T2D-DMPs at FDR 5% and the signal annotated to TXNIP. We averaged DNA methylation levels for the two bins that spanned each of the tested T2D-DMP base-pair location. The MeDIP-seq validation results are presented at nominal significance.

### Replication analysis

We pursued replication of the previously unreported T2D associated MCC-seq signals in an independent sample of 573 individuals from the TwinsUK cohort, with 33 T2D cases and 540 controls. The 573 participants had whole blood DNA methylation profiled using the same methylation profiling platform (MCC-seq). Quality control and analysis followed the same procedures as outlined for the discovery sample. To assess evidence for replication a Bonferroni threshold was applied to the number of tests (P = 0.05/20 ≈ 0.0025), and only results showing the same direction of the association as in the discovery sample were considered to replicate.

### Genetic data and meQTL analyses

To assess evidence for genetic impacts on DNA methylation levels we tested for genetic variants that were associated with DNA methylation at the T2D-DMPs (meQTLs). Genotype data were explored in 277 TwinsUK twin pairs from both the discovery and replication datasets with MCC-seq data, and were used for the identification of meQTLs. Genotyping of the full TwinsUK genetic dataset has been described previously.^45^ Briefly, genotyping was carried out using HumanHap300, HumanHap610Q, HumanHap1M Duo, and HumanHap1.2 M Duo 1 M arrays. Haplotypes were derived from pre-phasing using IMPUTE2 without a reference panel. Fast imputation was performed using these haplotypes and the 1000 Genomes phase 1 dataset. SNPs were excluded as part of quality control where they failed Hardy Weinberg equilibrium (P < 10^−6^), had a MAF <0.01, had missingness of more than 5% or an info score <0.8. In addition, individuals with discordant sex were removed. PLINK 2.0 was used to remove outliers in unrelated participants and GENESIS used for related participants. A deviation of more than 7 SD from the mean was considered an outlier. Pruning was undertaken to reduce relatedness, with participants with IBS >0.125 (calculated using PLINK 2.0) removed.

For identification of SNPs that were meQTLs a linear model was fitted using the MatrixEQTL R package,^46^ where the methylation was the response variable, and dosage of minor allele was the predictor. Covariates included age, sex, BMI, smoking status and blood cell composition as fixed effects. Both *cis* me-QTL and *trans* meQTL SNPs were included, where the *cis* interval was defined as ± 1 Mb from the CpG site. A stringent *cis* me-QTL P-value threshold was used to test for significance (P = 1 × 10^−5^), as previously described^47^ and in *trans* a stricter threshold was applied (P = 5 × 10^−8^). The most significantly associated SNP per CpG site was reported as the meQTL for the T2D-DMP. In addition, the GoDMC meQTL database^48^ based on the 450k array was also used to investigate meQTLs at the nearest CpG in the database.

### Enrichment in GWAS regions

We carried out an analysis to determine whether the epigenetic associations were enriched in regions previously found to be associated with T2D in genome wide association studies (GWAS). Firstly, we determined GWAS regions, which were defined as a 50 kb window around the lead SNPs identified as being associated with T2D in two recent large European ancestry GWAS studies.^49^^,^^50^ For the purpose of determining whether there was an enrichment of T2D-DMPs in GWAS regions, we utilised T2D-DMPs identified at a more relaxed threshold of FDR 50% in the case–control analysis (which resulted in 5057 T2D-DMP signals, around 1% of the total sites). We then assessed whether there was a difference in the number of CpG sites in and out of the identified GWAS regions. A Fisher's Exact Test was used to determine significance at a nominal threshold (P < 0.05). Secondly, we assessed whether the 20 genes to which the FDR 25% T2D-DMPs were annotated had been previously found in GWAS studies to have variants associated with T2D, its complications, metabolic health or inflammatory diseases. The GWAS catalog^51^ was used for this purpose.

### Metabolomic profiling

To assess the functional relevance of T2D-associated MCC-seq signals, we explored their blood metabolomic signature. Blood metabolomic profiles were analysed for 181 individuals in TwinsUK in both the discovery and replication samples, where metabolomic data were obtained in samples collected within 5 years of the MCC-seq sample data. Fasting serum metabolite levels for 592 metabolites were detected and quantified by Metabolon, Inc (Durham, USA), as described in detail previously.^52^ Briefly, the metabolomics analysis was carried out on a platform of four ultra-high performance liquid chromatography-tandem mass spectrometry (UPLC-MS/MS) instruments. Sample preparation used the MicroLab STAR system and samples underwent a multistep preparation process including removal of protein, division into five fractions for analysis using different methods and one backup, removal of organic solvent and storage overnight under nitrogen. Raw data underwent quality control using Metabolon's hardware and software. Metabolites included amino-acids, peptides, carbohydrates, energy intermediates, lipids, nucleotides, cofactors and vitamins, and xenobiotics. Linear models assessed the association between methylation levels at each T2D-DMP and blood metabolite levels. The linear model was similar to the model used in the main analysis, that is, methylation was the response variable and metabolite was the predictor. The model included covariates for smoking, age, BMI, family structure and cell counts, and an additional covariate reflecting the time difference between the time of blood draw for methylation profiling and for metabolomic profiling. Two Bonferroni multiple testing thresholds were assessed, the main focus being on FDR 5% significant CpGs (3 signals at P = 0.05/(592∗3) ∼ 2.8 × 10^−5^), and secondly, we also considered 20 FDR 25% significant CpGs (P = 0.05/(592∗20) ∼ 4.2 × 10^−6^).

### T2D complications

To assess the potential impact of T2D-DMPs on T2D progression and development of complications, we explored additional phenotype data in the TwinsUK discovery and replication samples. Individuals with T2D who had developed diabetic retinopathy were identified through self-reported questionnaire responses. The association between diabetic retinopathy and DNA methylation at chr14:77736811 (*NGB)* and chr14:57265055 (*OTX2)* was investigated in 9 T2D retinopathy cases (which included 4 individuals who were related as part of 2 twin pairs) and 66 T2D controls using a two-tailed t-test at a nominal significance threshold (P = 0.05).

Blood pressure measured at clinic visits was available for 77 individuals with T2D from the discovery and replication samples. For the purpose of this analysis, high blood pressure was defined as systolic pressure greater than 130 or diastolic pressure greater than 80. The association between DNA methylation at chr19:11529587 (*RGL3*) and high blood pressure in 77 T2D individuals was assessed using a two tailed t-test at a nominal significance threshold (P = 0.05).

We further explored both retinopathy and blood pressure in the MeDIP methylation data at the same sites using the same approach. In this non overlapping dataset there were 34 T2D retinopathy cases and 82 T2D controls and 110 individuals with T2D with blood pressure data.

### Role of funders

Funders had no role in the study design, data collection, data analyses, interpretation, or writing of the manuscript.

## Results

**Fig. 1:**
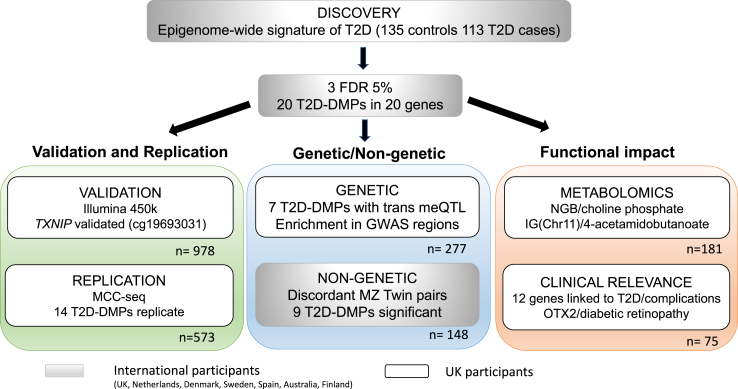
**Study design and results summary.** Epigenome-wide analysis of T2D in 113 T2D cases and 135 controls. Downstream analyses explored genetic-based and genetic-independent associations, functional follow-ups, and clinical relevance to T2D complications.

Associations between T2D and DNA methylation levels profiled using the MCC-seq platform were explored in 248 whole blood samples from 7 international twin cohorts in the discovery stage (135 controls and 113 T2D cases). Participants were predominantly of European ancestry with an average age of 60 ± 13 and mostly female (Table 1). The primary analysis sought to identify previously unreported sites of differential methylation associated with T2D (T2D-DMPs) in a T2D case–control analysis. Integrating genotype data along with analysis of 74 T2D-discordant MZ twin pairs, provided the opportunity to explore the genetic basis of T2D-DMPs, that is, distinguishing genetically-based from genetically-independent T2D associated differential methylation signals. The findings were subject to replication, validation, functional follow-up and enrichment analyses. Fig. 1 shows an overview of the study design.

### T2D case control epigenome wide signals

**Fig. 2:**
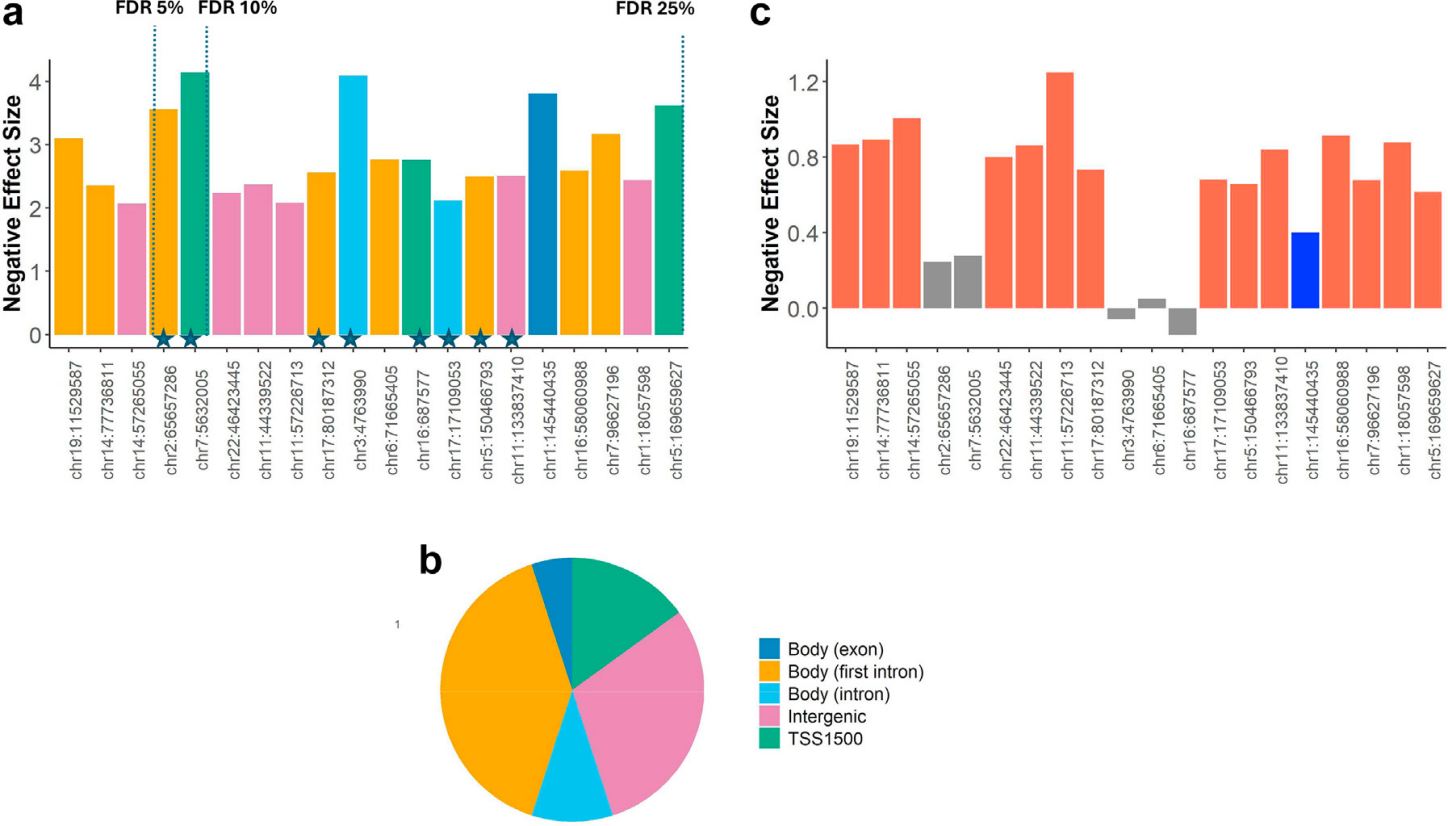
**Differential methylation signature of T2D in whole blood. a.** Differentially methylated sites ranked by FDR, showing effect size (y axis, expressed as negative effect size as all effects are hypomethylated in T2D) and genomic annotation (colour coded). T2D-DMPs in enhancer regions are indicated with a star. Vertical dotted lines show FDR 5%,10% and 25% thresholds. **b.** Proportion of T2D-DMPs in each genomic annotation category. **c.** Effect sizes in the replication sample, where results replicating at multiple testing threshold are shown in red, and results nominally significant are shown in blue.

**Fig. 3:**
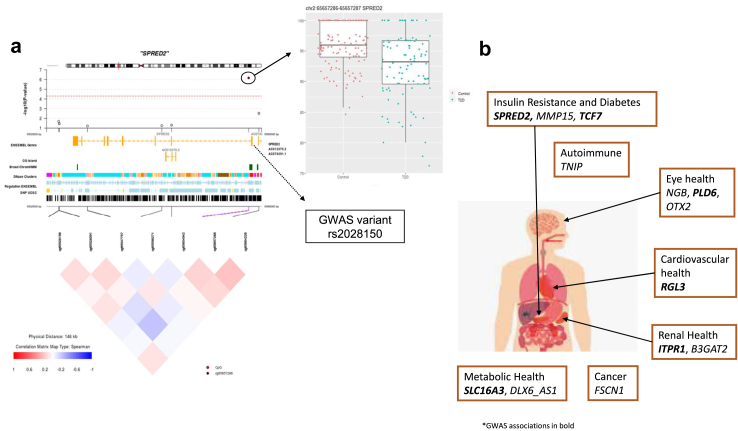
**Genes annotated to T2D-DMPs and their relevance to human health. a.** Epigenetic association between T2D and *SPRED2* DNA methylation displayed in a coMET plot,^34^ including T2D-methylation association results (top panel) along with functional annotation of the region (middle panel), and pattern of co-methylation at the CpG sites in the 450 k array annotated to *SPRED2* (bottom panel). Broad ChromHMM regions are displayed using UCSC genome browser colour schemes. GWAS variant rs2028150 is labelled and is located 2.27 kb from the MCC-seq T2D-DMP methylation site chr2:65657286. **b.** Biological relevance of genes annotated to FDR25% T2D-DMPs to human phenotypes, with published GWAS associations shown in bold.

A T2D case–control analysis, between 113 individuals with T2D and 135 individuals without T2D, identified 3 previously unreported genome-wide significant sites of differential DNA methylation annotated to the body of *RGL3* (chr19:11529587), the body of *NGB* (chr14:77736811) and downstream from *OTX2* (chr14:57265055) at FDR 5%. *RGL3* harbours genetic variants associated with blood pressure,^53^ *NGB* is a neuroprotective protein expressed strongly in the retina^54^ and *OTX2* is involved in the development of the eyes,^55^ and therefore the function of these genes suggests potential links to T2D complications. At FDR 25% altogether 20 differential methylation positions (T2D-DMPs) were identified (Table 2, Fig. 2a) in 20 genes (Table 2, Fig. 3). These 20 T2D-DMPs are previously unreported, with one around 1 kB from a previously identified T2D-associated CpG site in *TXNIP* (cg19693031). In addition to *TXNIP*, differential methylation has previously been found in regions annotated to *SPRED2* and *ITPR1.**^9^* All T2D-DMPs are hypomethylated in T2D with similar effect sizes (Table 2). Altogether 13 of the 20 T2D-DMPs are in gene promoters, with three in transcription start sites (chr7:5632005 *FSCN1*; chr16:687577 *METTL26*; chr5:169659627 *C5orf58*), and 8 of the 20 T2D-DMPs are located in enhancer regions (Fig. 2); suggesting a role of T2D-DMPs in gene regulation. Furthermore, twelve of the 20 T2D-DMPs are located in gene bodies, which is consistent with previous studies.^19^ All of the T2D-DMPs located in gene bodies are in intronic regions, with the exception of the signal in *TXNIP* (chr1:145440435). The T2D-DMPs are mainly located either in or near CpG Islands (Supplementary Table S9).

**Table 2:** Differentially Methylated CpG sites associated with T2D at FDR 25%.

Site	Gene	Position	Effect^b^	P-value	FDR	Replication effect	Replication P-value
chr19:11529587-11529588	*RGL3*	Body (first intron)	−3.10	1.83E-07	0.05	−0.867	6.59E-06
chr14:77736811-77736812	*NGB*	Body (first intron)	−2.36	2.55E-07	0.05	−0.893	1.16E-05
chr14:57265055-57265056	*OTX2*	Downstream	−2.07	2.94E-07	0.05	−1.007	8.36E-08
chr2:65657286-65657287	*SPRED2*	Body (first intron)	−3.56	7.03E-07	0.07	−0.244	2.30E-01
chr7:5632005-5632006	*FSCN1*	TSS1500	−4.14	7.32E-07	0.07	−0.278	1.44E-01
chr22:46423445-46423446			−2.24	1.80E-06	0.13	−0.800	3.19E-05
chr11:44339522-44339523			−2.37	1.91E-06	0.13	−0.861	9.83E-06
chr11:57226713-57226714	*RTN4RL2*	Upstream	−2.08	1.96E-06	0.13	−1.248	1.08E-11
chr17:80187312-80187313	*SLC16A3*	Body (first intron)	−2.56	2.67E-06	0.15	−0.732	2.62E-04
chr3:4763990-4763991^a^	*ITPR1*	Body (middle intron)	−4.09	3.67E-06	0.19	0.060	7.63E-01
chr6:71665405-71665406^a^	*B3GAT2*	Body (first intron)	−2.77	4.34E-06	0.19	−0.051	7.84E-01
chr16:687577-687578	*Ak201549/LOC10028175/METTL26*	TSS1500 (METTL26)	−2.76	4.45E-06	0.19	0.140	4.44E-01
chr17:17109053-17109054	*PLD6*	Body (intron)	−2.12	4.82E-06	0.19	−0.680	3.32E-04
chr5:150466793-150466794	*TNIP1*	Body (first intron)	−2.50	5.76E-06	0.20	−0.658	3.79E-04
chr11:133837410-133837411			−2.51	6.02E-06	0.20	−0.840	2.74E-05
chr1:145440435-145440436	*NBFF10/LOC100288142/TXNIP*	TXNIP (body exon) LOC/NBFF10 (intron)	−3.81	6.24E-06	0.20	−0.400	3.81E-02
chr16:58060988-58060989^a^	*MMP15*	Body (first intron)	−2.59	7.34E-06	0.22	−0.915	2.41E-06
chr7:96627196-96627197^a^	*DLX6-AS1*	Body (first intron)	−3.17	9.16E-06	0.24	−0.679	8.06E-04
chr1:18057598-18057599			−2.44	9.19E-06	0.24	−0.879	3.71E-06
chr5:169659627-169659628^a^	*C5orf58*	TSS1500	−3.62	9.44E-06	0.24	−0.615	2.30E-03

a This CpG site is in the 450 k array.

b Effect size is shown without normalisation.

Gene set enrichment analysis (GSEA) of the genes that the FDR 25% T2D-DMPs annotate to was carried out in the Human Molecular Signatures Database (MSigDB), and identified a significant (FDR <5%) enrichment for two sets of genes. The first set included genes upregulated in CD4+ T cells overexpressing *FOXP3* and *PPARG1* (4 genes). The second set included genes, which contained binding sites for the transcription factor CHAF1B within their promoter sequence (6 genes, including *FSCN1*, *TXNIP*, *ITPR1*, *SLC16A3*, *SPRED2* and *METTL26*).

### Sensitivity analyses including BMI

BMI is a major risk factor for T2D, therefore, we assessed its impact by including it as a covariate in sensitivity analyses. In analyses incorporating BMI the resulting T2D-DMP P-values and effect sizes were similar to the primary analysis excluding BMI, however, the strength of association was attenuated (Supplementary Table S3). Nine of the 20 T2D-DMPs still met the FDR 25% threshold including the three original FDR 5% results (now at FDR 9% when BMI is included in the model). Two additional sites met the FDR 25% threshold including chr5:133449700 (*TCF7*) and chr15:58085147 (intergenic). *TCF7* plays a role in beta cell function,^56^^,^^57^ activates immune system genes, and genetic variants in this gene are associated with Type 1 Diabetes.^58^

### Validation of T2D methylation signature

We sought to validate the peak MCC-seq T2D-DMPs using whole blood DNA methylation profiled on the 450k array platform, as well as a subset of the peak T2D-DMPs using the MeDIPseq platform. In the 450k validation, altogether 978 participants from TwinsUK were selected for validation, and these included 44 individuals (19 cases and 25 controls) from the primary analysis. The validation analysis tested the association between DNA methylation with both T2D status (49 cases and 681 controls) and with fasting blood glucose (FBG; 978 individuals). There was an exact overlap between the MCC-seq T2D-DMPs CpG site and the 450 k array CpG site for 5 T2D-DMPs, while the remainder of the MCC-seq T2D-DMP sites were not profiled on the 450 k array. In this instance we used the nearest 450 k CpG site up to a distance of 2.7 kB. Site cg19693031 (in *TXNIP*) validated with significant association at Bonferroni multiple testing threshold with both T2D (P = 3.4 × 10^−8^) and FBG (P = 2.7 × 10^−7^), with a consistent direction of association. Two further sites showed nominal significance in the FBG analysis, including cg15989436 *(TNIP1;* P = 0.0074) and cg01088070 (*B3GAT2;* P = 0.0268) (Supplementary Table S2). In addition, whilst not meeting nominal significance, 10 further T2D-DMPs showed the same direction of effect in the T2D association analysis (in cg02985292 (*METTL26*), cg01088070 (*B3GAT2*), cg10256249 (*SPRED2*), cg27175093 (*OTX2*), cg15989436 (*TNIP*), cg05468064 (chr22:46423445), cg21545390 (*DLX6*), cg15131789 (chr1:18057598), cg02400572 (*NGB*), cg08995368 (*FSCN1*)) and 2 further T2D-DMPs matched direction of effect in only the FBG analysis (in cg21545390 (*DLX6*), cg15131789 (chr1:18057598)).

Validation analyses were also carried out using MeDIP-seq data. In the MeDIPseq validation 3434 participants from TwinsUK (116 cases and 3318 controls) were selected for validation, and these did not include any participants in the primary analysis. Validation was tested for *RGL3* (chr19:11529587), *NGB* (chr14:77736811), *OTX2* (chr14:57265055), and *TXNIP* (chr1:145440435-145440436). Direction of effect matched across MCC-seq and MeDIP-seq data for all signals tested, and *NGB* (chr14:77736811) showed nominally significant results (Supplementary Table S11).

### Replication of T2D methylation signature

The 20 FDR 25% T2D-DMPs were evaluated for replication in an independent blood MCC-seq methylation sample from TwinsUK. Of the 20 FDR 25% T2D-DMPs reported in this study 19 are previously unreported and only the 450 k CpG site in *TXNIP* (cg19693031; 1.1 kb away from MCC-seq T2D-DMP chr1:145440435-145440436) has been robustly replicated in previous studies. Here, we pursued replication of all 20 T2D-DMPs in an independent sample of 573 TwinsUK participants profiled using MCC-seq, including 33 individuals with T2D at the time of blood collection. Fourteen signals replicated at a Bonferroni corrected threshold (P = 0.0025) and 1 further signal replicated at nominal significance (P < 0.05; Fig. 2c) and with a consistent direction of association. The 14 signals included all three FDR 5% results (*RGL3*, *NGB* and *OTX2*) and signals annotated to *RTN4L2*, *SLC16A3*, *PLD6*, *TNIP*, *MMP15*, *DLX6-AS1, C5orf58* and 4 further intergenic regions (Chr22:46423445, Chr11:44339522, Chr11:133837410, Chr1:18057598) (Supplementary Table S4).

DNA methylation is a potential mechanism for mediating genetic and environmental impacts on T2D, or the differential methylation signals observed could be secondary to disease. The next series of analyses consider this in more detail, exploring putative non-genetic and genetic sources of variation at the observed T2D methylation signature.

### Genetic independent (MZ twin pair) analysis

Using a discordant MZ twin pair model including 74 MZ twin pairs discordant for T2D, we explored whether the T2D-DMPs showed genetically independent effects by contrasting DNA methylation profiles between genetically identical T2D-discordant twins. Genetically independent sites of differential methylation likely reflect environmental effects on T2D and/or may arise as a consequence of T2D. These signals can also be informative for T2D disease progression and development of T2D complications. A t-test of the methylation residuals, allowing for covariates, showed that all of the 20 T2D-DMPs except one (intergenic site on chr11:133837410) were nominally significant and with the same direction of effect (Supplementary Table S1) in the genetically independent analyses. Of the 20 FDR 25% tested T2D-DMPs, 9 sites (in *OTX2*, *SPRED2*, *SLC16A3*, *B3GAT2*, *METTL26*, *TNIP1, TXNIP, MMP15* and an intergenic regions) had significant associations in the T2D-discordant MZ twin analysis after multiple testing correction (Supplementary Table S1). These target genes with a range of functions including regulating growth (*SPRED2*), catalysing lactic acid and pyruvate transport (*SLC16A3*, a member of the MCT family), catalysing glycosaminoglycan metabolism (*B3GAT2*) and autoimmunity (*TNIP1*). *METTL26* function is unknown, however, it has been observed to be expressed at a high level in cancerous cells and associated with poor prognosis.^59^ In addition to the 20 T2D-DMPs the two sites identified in the BMI sensitivity analysis (chr5:133449700 (TCF7) and chr15:58085147 (intergenic)) were also nominally significant (P < 0.05) for genetically independent effects.

### Genetic variation underlying T2D methylation signatures

We assessed if the differential methylation signature of T2D may be influenced by genetic variants, or DNA methylation quantitative trait loci (meQTLs). Using two approaches we observed evidence for a genetic basis at 8 of the 20 FDR 25% sites when considering only the target MCC-seq T2D-DMP CpG site, and at 15 of the 20 FDR 25% sites when considering the target CpG or the nearest 450 k CpG site. First, we explored the association between genetic variation and DNA methylation levels at the 20 T2D-DMPs in 277 twin families from TwinsUK. We identified 7 T2D-DMPs with significant *trans* meQTL associations (P = 5 × 10^−8^, Supplementary Table S5) in *NGB, OTX2, SLC16A3, ITPR1, TNIP1*, and two intergenic regions (chr1:18057598 and chr11:44339522). The most significant association was between a genetic variant in the *HPN* gene (chr19:35561358) and DNA methylation in *ITPR1* (chr3:4763990-4763991). *ITPR1* has been shown to have a role in diet induced diabetes^60^ and *HPN* encodes hepsin and it has been shown in mouse models that hepsin deficiency lowers blood glucose.^61^

**Fig. 4:**
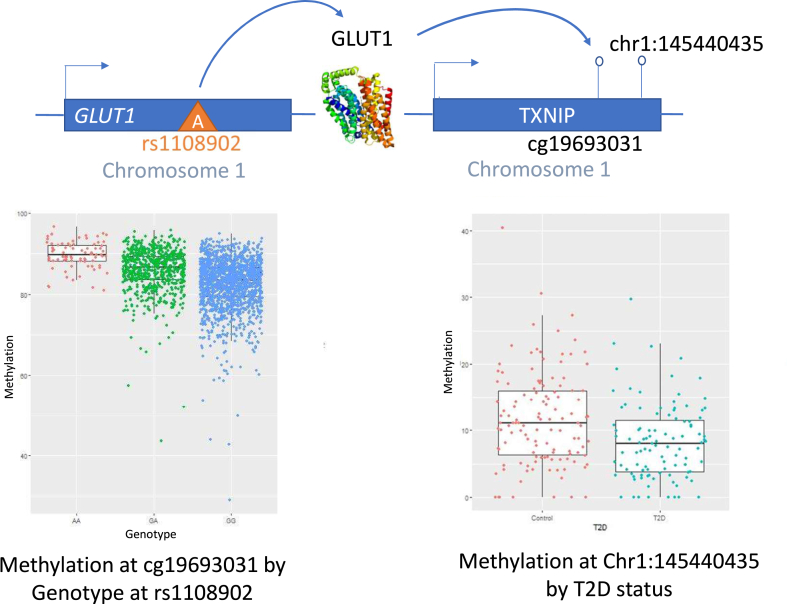
**DNA methylation in *TXNIP* is under distal genetic control.** SNP rs1108902 in *GLUT1* is a trans-meQTL for DNA methylation levels at CpG site cg19693031 in *TXNIP,* located around 1 kb away from the MCC-seq T2D-DMP on chromosome 1 (chr1:145440435). Shown are boxplots of the variation in DNA methylation levels by T2D status at chr1:145440435 in *TXNIP* for the discovery cohort, and change in *TXNIP* cg19693031 DNA methylation levels by genotype at rs1108902 in *GLUT1* in TwinsUK participants.

Second, using the GoDMC meQTL database^48^ we investigated meQTLs for the target CpG T2D-DMP site, if this was profiled on the 450k array, or for the nearest 450k CpG site to the T2D-DMP using the same thresholds. Five of the 7 T2D-DMP CpGs identified above also had evidence for a genetic basis in GoDMC, and a further 8 sites were not profiled on the 450k array, but the nearest 450k CpG had meQTLs in GoDMC. Altogether these 13 450k CpGs had GoDMC meQTLs in *cis* only (11), *trans* only (1), or both *cis* and *trans* (1) (Supplementary Table S6). There were 147 *trans* meQTLs for cg19693031 in *TXNIP,* of which 133 are in *SLC2A1* (*GLUT1*) which is a major glucose transporter located over 100 MB away from *TXNIP* (Fig. 4). *TXNIP* expression causes a reduction in GLUT1,^62^ which in turn transports more blood sugar when blood glucose is high. Suppressing *GLUT1* has been shown to reverse high retinal blood glucose levels in diabetic mice.^63^ The remaining SNPs are in a 125 kB window of *SLC2A1*.

Of the 8 T2D-DMPs that had direct evidence for a genetic basis of the target MCC-seq CpG site, five (in *OTX2, SLC16A3, B3GAT2, TNIP1* and intergenic region chr11:44339522) were also identified in the genetically-independent results from the T2D-discordant MZ twin analysis. Thus, both genetic and non-genetic effects contribute to DNA methylation variation at these 8 T2D-DMPs. It is therefore likely that DNA methylation levels at these five signals reflect consequences of T2D, which may potentially be risk or effects of developing T2D complications.

### Enrichment of T2D methylation signature in GWAS regions

Previous studies have found that there is an overlap between T2D GWAS results and T2D-DMPs.^19^ Here, we also find an enrichment of T2D-DMPs in T2D GWAS regions based on two different approaches. First, we tested whether there was an enrichment of FDR 25% T2D-DMPs in T2D GWAS regions (defined as a window of 50 kb either side of a lead SNP in recent large GWAS). One of the 20 T2D-DMPs in *SPRED2* is in a T2D GWAS region (Fig. 3a), as well as one of the T2D-DMP results with BMI as a covariate (in *TCF7*). The differential methylation in *SPRED2* (chr2:65657286) and the GWAS variant (rs2028150) are located in the same first intron region of the gene.

Due to the small number of T2D-DMPs at FDR 25% we also explored GWAS enrichment for T2D-DMPs at a more relaxed FDR 50% threshold, resulting in 4452 T2D-DMPs. We also found a significant enrichment of these FDR 50% T2D-DMPs in previously published T2D GWAS regions (Fisher Exact Test P = 3.5 × 10^−8^, Supplementary Table S8).

Second, we assessed if the genes to which the FDR 25% T2D-DMPs were annotated contained genetic variants that had previously been associated with either T2D or related phenotypes in GWAS studies. We found GWAS links between 7 T2D-DMP genes and T2D or related phenotypes (Fig. 3b), including *SPRED2* (T2D), *ITPR1* and *PLD6* (Diabetic complications), *SLC16A3* (BMI), *TCF7 (*Type 1 Diabetes*), RGL3* (blood pressure) and *TNIP1* (autoimmune traits).

### Metabolomic follow-up of T2D methylation signature

**Fig. 5:**
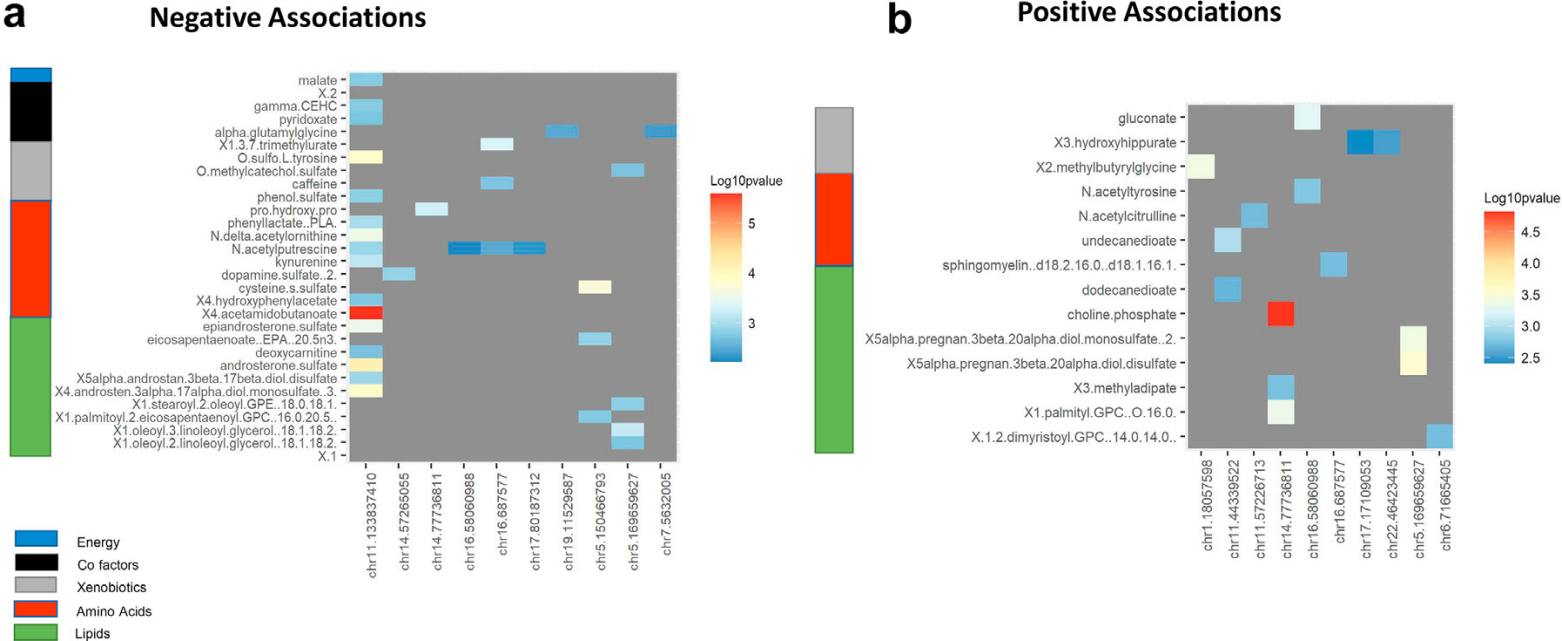
**Association between T2D methylation signature and metabolomics.** Heatmap showing significant T2D-DMP metabolite associations (FDR <5%) and their effect size. Only significant (a) negative and (b) positive associations are shown, with grey areas reflecting correlations that did not meet the multiple testing threshold, and T2D-DMPs not shown where there were no significant associations (FDR <5%).

To explore the functional relevance of the T2D-associated differential methylation variation, we tested the association between DNA methylation levels at the 20 T2D-DMPs and blood metabolites. Circulating blood metabolites included amino acids, peptides, carbohydrates, energy intermediates, lipids, nucleotides, cofactors and vitamins, and xenobiotics. Of the 592 metabolites tested, two were significantly associated with DNA methylation levels at T2D-DMPs at a Bonferroni multiple testing threshold (Fig. 5, Supplementary Table S7). The two signals included choline phosphate levels, which were associated with methylation in *NGB* (chr14:77736811, P = 1.5 × 10-5). Choline phosphate is a reactant in the formation of citicoline. There was a positive association between methylation levels and choline phosphate, with a negative association found between DNA methylation and T2D. There is evidence that citicoline use can alleviate symptoms of diabetic complications, including in diabetic retinopathy and diabetic neuropathy.^64^^,^^65^

The second signal was in 4-acetamidobutanoate, where metabolite levels were associated with DNA methylation in an intergenic region (chr11:133837410, P = 2.6 × 10-6). Metabolite 4-acetamidobutanoate is a precursor to ornithine, a component of the urea cycle.^66^ Previous work has shown 4-acetamidobutanoate to be the most enriched urinary metabolite when individuals with low liver and kidney disease severity were compared with those with high disease severity.^67^

### T2D methylation signature and diabetic complications

A systematic analysis of the association between DNA methylation and T2D complications has not been thoroughly explored to date. In this study, the 3 T2D-DMPs at FDR 5% annotated to genes which have biological relevance to T2D complications, and metabolomic follow-up strengthens the link to T2D complications. Therefore, we further explored links between T2D-DMP DNA methylation levels and targeted T2D complications, specifically, diabetic retinopathy and high blood pressure.

Both *NGB* and *OTX2* have biological links to eye health, therefore we focused on investigating the association between DNA methylation at these two genes and diabetic retinopathy status in T2D cases. We explored DNA methylation levels at chr14:77736811 (*NGB)* and chr14:57265055 (*OTX2)* in 9 T2D retinopathy cases and 66 T2D retinopathy controls, selected from the TwinsUK discovery and replication T2D cases. A nominally significant difference in DNA methylation was observed at chr14:57265055 in *OTX2* (P = 0.025), but not at chr14:77736811 in *NGB* (P = 0.62). We did not observe a nominally significant difference in the MeDIPseq data for chr14:57265055 in *OTX2*, but both sites showed consistent direction of effects. That is, we observed lower DNA methylation levels in T2D cases with diabetic retinopathy compared to T2D cases without retinopathy, and this was consistent with the results from the MCCseq analysis.

Genetic variation in *RGL3* has previously been associated with blood pressure variation (Wang and Wang, 2019). We therefore investigated the association between DNA methylation at chr19:11529587 (*RGL3*) and blood pressure levels in 77 T2D cases from the TwinsUK discovery and replication sample, but did not observe a nominally significant difference. We also did not observe a significant difference in the MeDIPseq data.

## Discussion

In this study we sought to apply a DNA methylation profiling approach targeting functional regions of the genome (MCC-seq) in whole blood to identify previously unreported CpG sites that were significantly differentially methylated with T2D and assess their functional significance and biological relevance for T2D complications. Participants in the study were selected from 7 international twin cohorts and included the largest number of T2D-discordant MZ twins pairs studied to date. We identified and replicated 3 FDR 5% signals for T2D in 248 discovery phase participants (113 T2D cases, 135 controls) and 573 replication phase participants (33 T2D cases, 540 controls; 978 individuals with FBG). Altogether 20 CpG sites in 20 genes were differentially methylated at a more relaxed FDR 25%, of which 14 replicated. These 20 sites were then analysed in 74 T2D-discordant MZ twin pairs. We identified genetic-independent effects at 9 T2D-DMPs, which were significantly differentially methylated within T2D-discordant MZ twin pairs. At these sites DNA methylation are likely either consequences of T2D, or they are mediating the effects of non-genetic T2D risk factors. We further assessed differential methylation for links to genetic variation, testing for enrichment in GWAS regions, comparing genes to which the T2D-DMPs were annotated to results from GWAS, and investigating genetic variants influencing methylation levels. We found many links to genetic associations with T2D and related phenotypes, highlighting the strong genetic contribution both to methylation levels and to T2D. A subset of five signals, including *OTX2*, had both a robust genetic basis and also showed differences in T2D-discordant MZ twins, suggesting that these signals may be secondary to T2D and could potentially reflect development of T2D complications. We further found strong links between the T2D-DMP genes identified in our study and T2D complications, which were in part supported in metabolomic follow ups. Further analyses in a sample subset identified a nominally significant difference in methylation at one of the FDR 5% T2D-DMPs in *OTX2* among individuals with T2D, between those with diabetic retinopathy and those without.

Using the MCC-seq panel we were able to target around 3.7 million CpG sites and selected sites that showed the most variation in methylation levels between individuals for further analysis. Additionally, this panel was designed to target functional regions of the genome.^28^ This resulted in the identification of 20 previously unreported T2D-DMPs including one CpG site in *TXNIP,* which was near to a previously identified T2D-associated CpG site. Of the 20 T2D-DMPs, 8 were located in the first intron of the gene body. Previous work^68^ has shown a consistent correlation across both tissues and species between DNA methylation at the first intron and gene expression, indicating that the methylation changes in these sites may also be linked to changes in gene expression. Furthermore, 8 of the 20 T2D-DMPs were located in enhancer regions suggesting potential regulatory impacts. Three of the 20 T2D-DMPs reached genome wide significance at FDR 5% and were located in genes with possible links to diabetic complications such as blood pressure, cardiovascular disease and diabetic retinopathy (*RGL3*, *NGB and OTX2*). *RGL3* has been shown to be a potential effector of MRas and Ras.^69^ The *MRAS* gene is involved in a number of cell processes including signal transduction and cell growth and is suggested to have a key role in cardiovascular function.^70^ Additionally, *RGL3* contains a significant number of SNPs which have been found to be associated with blood pressure.^53^ *NGB* encodes a relatively recently discovered neuroglobin which is a neuroprotective protein for the retinal ganglion cells.^54^ This protein is strongly expressed in the retina and has been shown to have a protective effect against damage due to high pressure in the eye.^71^ It is unclear how this relates to diabetic retinopathy, which is caused by glucose damage to blood vessels in the retina, but a loss of methylation and a potential upregulation of NGB during diabetes suggests that more neuroprotective protein is produced in T2D cases in response to the high levels of blood glucose. Furthermore, our follow-up metabolomic analysis found a significant association between *NGB* methylation and levels of choline phosphate in serum. Choline phosphate is used to form citicoline which has been shown in combination with vitamin B12 to stabilize and decrease functional impairment in diabetic retinopathy.^65^ It has also been found to be effective in reducing diabetic neuropathic pain.^64^ A significant metabolic association was also found with 4-acetaminobutanoate and methylation at an intergenic site. Metabolite 4-acetaminobutanoate is a precursor to the urea cycle and has been previously linked with kidney health.^67^ *OTX2* is also related to eye health and an increase in *OTX2* levels in mouse models has been shown to promote the survival of retinal ganglion cells, the loss of which leads to visual impairment in diabetic retinopathy.^72^ Further, we found a nominally significant difference in *OTX2* DNA methylation between individuals with diabetic retinopathy and individuals with diabetes who did not have retinopathy based on a limited sample size. Lastly, gene set enrichment analysis of the genes annotated to the T2D-DMPs identified an enrichment of target genes of binding sites for the transcription factor CHAF1B, which is required for haematopoiesis.^73^ Haematopoiesis dysregulation is a consequence of diabetes, suggesting further links between our T2D-DMPs and T2D complications.^74^

The next most significant result was in *SPRED2* where we observed significant differential methylation within 2.5 kB of a SNP (rs2028150), which has been previously associated with T2D in GWAS.^51^ This SNP is also associated with levels of methylation in *SPRED2* in GoDMC datasets,^48^ along with many other SNPs in the same gene, which could suggest that the differential methylation we observe is due solely to genetic variation between individuals with diabetes and those without. However, we additionally found that the *SPRED2* methylation signal remained significant in the T2D-discordant MZ twin analysis, suggesting that there are both genetic and non-genetic influences on the T2D differential methylation signal in this gene. This is of particular note due to *SPRED2* having been identified as a potential therapeutic tool for the prevention of insulin resistance.^75^ A knockout mice study showed that removal of *SPRED2* increased insulin resistance from a high fat diet suggesting *SPRED2* to be a negative regulator of insulin resistance *via* its suppression of the ERK/MAPK pathway.^75^

Almost all the remaining T2D-DMPs annotated to further genes of interest with either functional effects potentially explaining increased propensity of T2D cases for complications, or highlighting genes with a role in metabolic health. Genes linked to T2D complications or comorbidities include *FSCN1*, *ITPR1*, *B3GAT2* and *PLD6*. There is a positive association between diabetes and a number of cancers^76^ and we observed differential methylation in the transcription start site of *FSCN1,* which has been shown to be upregulated in cancer.^77^ *B3GAT2* encodes a protein which catalyses the formation of glycosaminoglycan-protein linkage. Abnormalities in glycosaminoglycan metabolism have been previously found in people with diabetes^78^ with a study in animal models showing that administration of glycosaminoglycans prevents deterioration in renal health in diabetic rats and reverts established lesions^79^. Diabetes is one of the leading causes of chronic kidney disease with people with diabetes at increased risk of end stage renal disease. The differential methylation is observed in our T2D-discordant analyses and also *B3GAT2* is associated with obesity related traits in GWAS studies^51^ suggesting possible genetic and environmental contributors to disease relating to this gene. Some T2D-DMP genes show a link to both T2D complications and genetic associations to T2D or BMI. These include *ITPR1* which has been shown to have a role in both the development of diet induced diabetes^60^ and a genetic association with kidney disease. Previous GWAS studies have also found associations between variants in *PLD6* and both BMI and diabetic retinopathy.^51^

Genes with links to metabolic health include *SLC16A3*, *DLX6-AS1* and *MMP15*. *SLC16A3* (also known as *MCT4*) is a member of the monocarboxylate transporter family and has a role in the movement of lactate across the plasma membrane. Lactate is an intermediate metabolite of glucose, and an excess of lactate has been shown to be a risk factor for insulin resistance.^80^ *SLC16A3* was also observed to be differentially methylated in the genetically identical discordant twin pairs analysis, suggesting that this methylation signal may be partly environmentally driven or secondary to T2D onset. *DLX6-AS1* is part of a complex that activates transcription of Dlx-5 and Dlx-6. Mouse models show that expression of these genes in GABAergic neurons has significant effects on metabolism, healthy ageing and longevity.^81^ Finally, there is evidence that *MMP15* gene expression alters in the development of insulin resistance.^82^

We observed an overlap between the genes annotated to T2D-DMPs and genes identified in GWAS, in line with previous work.^19^ The enrichment of T2D-DMPs in T2D GWAS regions was also observed at a slightly more relaxed P-value threshold of FDR 50% indicating it is not only the most significant results that overlap. This supports previous work indicating that the genes that the twin-based T2D-DMPs identify have biological relevance to T2D, where some of these effects are detected at the level of genetic variation in these genes.

We validated one of the T2D-DMPs (chr1:145440435 in *TXNIP*) in the 450k array in a partially independent sample, observing significant differential methylation at cg19693031 associated with both T2D and FBG. This site has been previously reported and replicated as a differentially methylated signal for T2D^9^ and is one of the strongest T2D methylation signals independent of BMI (replicated in 5 of 10 blood methylation studies to date^9^). Recent large-scale analyses from over 27,000 samples within the GoDMC study^48^ identified many genetic variants influencing DNA methylation levels on the 450k array, and we used this GoDMC results database to investigate genetic variation influencing *TXNIP* methylation levels. We found that genetic variation at 133 different SNPs in *GLUT1* is associated with methylation levels in *TXNIP*. *TXNIP* expression causes a reduction in GLUT1, which in turn transports more blood sugar when blood glucose is high. A knock down of GLUT1 in mice using siRNA has been shown to reduce retinal blood glucose in diabetic mice to the level of non-diabetic mice providing the potential for mitigating diabetic retinopathy.^63^ GoDMC datasets in over 30,000 samples show that genetic variation in *GLUT1* affects the level of methylation observed in *TXNIP.* As such *TXNIP* methylation levels may provide an indicator for how well the GLUT1 transporter is functioning and potentially identify individuals who are susceptible to T2D complications. *TXNIP* hypomethylation with T2D also shows parallels with *AHRR* hypomethylation in response to smoke exposure. In both cases upregulation of the gene inhibits the body's ability to detoxify. TXNIP binds to thioredoxin (Trx) inhibiting its ability to neutralise oxidants, and prolonged overexpression of *TXNIP* has been shown to lead to premature cell death in diabetic retinopathy.^83^

There are several limitations to this study. The MCC-seq read depth cut-off of 5 reads is relatively low. On the other hand, the average read depth for the T2D DMP signals identified is around 20 or greater at most signals (Supplementary Table S10). A relatively low read depth threshold could lead to a greater false positive rate and lower accuracy compared to previous microarray based studies. Whilst the number of participants is larger than many DNA methylation studies of T2D and includes the largest number of T2D-discordant MZ twins, the sample size is still relatively modest and provides only moderate power to detect the small changes in methylation associated with the disease. Sex-specific effects were not explored due to the relatively modest sample size. The participants are nearly all of European ancestry and the results may not translate to other ethnicities. This study included only DNA methylation and not gene expression follow-ups. We are therefore not able to determine whether the differential methylation we observe also has an impact on gene expression, however, we were able to integrate circulating metabolic levels as a functional follow up. DNA methylation is a dynamic marker, and longitudinal studies are needed to explore the longitudinal stability, or potential reversibility, of the identified T2D-DMPs. Such longitudinal studies would be particularly beneficial in diseases like T2D, which progress over time and result in risk of developing clinical complications, which increases with longer periods of sustained high blood sugar. It would also be insightful to study methylation profiles in individuals whose blood glucose levels normalise following lifestyle interventions, in contrast to those where T2D progresses.

In conclusion, we observe a number of previously unreported CpG sites differentially methylated with T2D, which are annotated to genes with functional links to diabetic health. There is a strong interlink between genetics and methylation, and it is likely that most T2D associated DNA methylation signals arise as a consequence of disease because a number of sites are in genes linked to T2D complications or consequences of high blood glucose. Methylation levels at these sites may be potential biomarkers of disease progression and may help to understand mechanisms for the development of complications.

## Contributors

JTB and TDS designed the study. EG oversaw MCC-seq methylation profiling. CC led the data analysis with major inputs from LP and TM, and further support from SVM, JEC-F, MM, PC-T, SW, SC and PC. JTB, TDS, JO, OM, PS, KM, KHP, MO, CD, KK, KC, DB, GW, JvD, PM, NP, SW, CM, JT contributed samples and/or data. CC and JTB wrote the manuscript. All authors read and approved the manuscript. JTB and TM verified the underlying data.

## Data sharing statement

Discovery phase DNA methylation profiles with the exception of Danish cohort data are available from the European Genome-phenome Archive (EGA; Accession no. EGAD50000000286). Covariate and phenotype data are available upon request from each twin cohort as described below.

TwinsUK: Additional individual-level data are not permitted to be publicly shared or deposited due to the original consent given at the time of data collection, where access to these data is subject to governance oversight. However, these data can be applied for through a data access application, where all data access requests are overseen by the TwinsUK Resource Executive Committee (TREC). For information on access to these genotype and phenotype data and how to apply see https://twinsuk.ac.uk/resources-for-researchers/access-our-data/

Older Australian Twins Study: Data provided by the participants can be shared with other research groups by application to the CHeBA Research Bank and scientific review by the OATS Governance Committee (see https://cheba.unsw.edu.au/research-projects/older-australian-twins-study). A data application form can be obtained by emailing CHeBAData@unsw.edu.au.

Finnish Twin Cohort Study: Data are deposited with the Biobank of the Finnish Institute for Health and Welfare (https://thl.fi/en/web/thl-biobank/for-researchers/sample-collections/twin-study) with identification number THLBB2021_001. Data are available upon request, after approval of application by the biobank. For details on accessing the data, see https://thl.fi/en/web/thl-biobank/for-researchers/application-process.

Netherlands Twin Register: Data are available upon request, after approval of application by the NTR Data Access Committee. For information on procedures please see https://tweelingenregister.vu.nl/information_for_researchers/information-for-researchers and (https://ntr-data-request.psy.vu.nl/).

Murcia Twin Registry: Data are available upon request, after approval of application by the Murcia Twin Registry Committee. Information on data access procedures can be obtained from geminis@um.es.

Danish Twin Registry: Individual-level data are not permitted to be publicly shared or deposited due to the original consent given at the time of data collection, as well as GDPR. All data access requests are overseen by the Danish Twin Research Center. For information on access to data and how to apply, see https://www.sdu.dk/en/om_sdu/institutter_centre/ist_sundhedstjenesteforsk/centre/dtr/researcher.

Swedish Twin Registry: As a national research infrastructure the STR is open to applications also from abroad. Confidentiality review determines data access. However, linkage of additional personal data to large data repositories such as European Genome-phenome Archive (EGA) is generally not permitted. For information about how to apply, see https://ki.se/en/research/the-swedish-twin-registry.

## Declaration of interests

The authors have no conflicts to declare.

## Appendix A Supplementary data

Supplementary Tables

Supplementary Note

STREGA final

## Footnotes

Appendix A Supplementary data related to this article can be found at https://doi.org/10.1016/j.ebiom.2024.105096.
