# Supplementary material for: Enhanced resolution profiling in twins reveals differential methylation signatures of type 2 diabetes with links to its complications
Source: eBioMedicine. 2024 Apr 4;103:105096. doi: 10.1016/j.ebiom.2024.105096 (PMC11004697; doi:10.1016/j.ebiom.2024.105096)
Supplement: Supplementary Note [file mmc2.docx]

**Supplementary Note: Christiansen et al.**

## Study Cohorts and Participants

**TwinsUK**

TwinsUK is the largest twin registry in the UK with over 14,000 identical and

non-identical same-sex twins (age 16-100). The registry was established in 1992 and is one of the most detailed studies of adult twins in the world. Extensive data are available for over a thousand phenotypes, environmental exposures, lifestyle and social factors. Biological samples and data are available in a large subset of cohort participants. The cohort and data collected have been previously described (Verdi et al, 2019).

All research participants have signed informed consent prior to taking part in any research activities. Ethical approval was granted by the National Research Ethics Service London-Westminster, the St Thomas' Research Ethics Committee (REC reference numbers: EC04/015 and 07/H0802/84).

*Cohort-specific Acknowledgments*

**TwinsUK is funded by the Wellcome Trust, Medical Research Council, Versus Arthritis, European Union Horizon 2020, Chronic Disease Research Foundation (CDRF), Zoe Global Ltd and the National Institute for Health Research (NIHR) Clinical Research Network (CRN) and Biomedical Research Centre based at Guy’s and St Thomas’ NHS Foundation Trust in partnership with King’s College London. This work was supported by the European Research Council (ERC 250157 to TDS), the UK Biotechnology and Biological Sciences Research Council (BBSRC) and Economic and Social Research Council (ESRC) (BBSRC/ESRC ES/N000404/1 to JTB), and the JPI ERA HDHL DIMENSION grant (BBSRC BB/S020845/1 to JTB).**

*Determination of Type 2 Diabetic Status*

**An individual was categorised as T2D if either there were two or more indicators of T2D or T2D medication was listed in regular medications taken. Indicators included fasting blood glucose levels greater than 7 mmol/L and reporting of T2D incidence. Individuals who were insulin resistant, but not Type 2 Diabetic were excluded from the analysis as were individuals with only one indicator of T2D.**

**The Older Australian Twins Study**

**The Older Australian Twins Study (OATS) was designed to study healthy brain ageing in twins aged 65 and over who were recruited from the Australian Twin Registry and also via a recruitment drive. In the original OATS cohort, at baseline there were 623 twins with a mean age of 70.8 years and 65% were women. The participants were followed over time to assess both psychological and physical health. Fasting bloods were collected for biochemistry and genetic/epigenetic analyses.** Written informed consent was obtained from all participants and the ethics committees of the Australian Twin Registry, University of New South Wales, University of Melbourne, Queensland Institute of Medical Research and the South-Eastern Sydney and Illawarra Area Health Service (HREC: HC220479). **For further details of recruitment and data collection, please see Sachdev et al. (2009).**

*Cohort-specific Acknowledgments*

**We acknowledge the contribution of the OATS research team (https://cheba.unsw.edu.au/project/older-australian-twins-study) to this study. The OATS study has been funded by a National Health & Medical Research Council (NHMRC) and Australian Research Council (ARC) Strategic Award Grant of the Ageing Well, Ageing Productively Program (ID No. 401162); NHMRC Project (seed) Grants (ID No. 1024224 and 1025243); NHMRC Project Grants (ID No. 1045325 and 1085606); and NHMRC Program Grants (ID No. 568969 and 1093083). We thank the participants for their time and generosity in contributing to this research. This research was facilitated through access to Twins Research Australia, a national resource supported by a Centre of Research Excellence Grant (ID No. 1079102) from the National Health and Medical Research Council.**

*Determination of Type 2 Diabetic Status*

**Classification of T2D was defined as self-report of a diagnosis by a doctor, current usage of diabetes medication or a fasting blood glucose ≥7.0mM**

**The Finnish Twin Cohort Study**

**The Finnish Twin Cohort Study (FTCS) consists of three separate cohorts established for different birth years. The current study included twin pairs from the older cohort, which was established in the 1970s with 13,888 twins born before 1958, and from a younger cohort, FinnTwin16, established to carry out longitudinal studies of adolescent twins and their families (twins born between 1975 and 1979).Extensive longitudinal phenotype data is available from the participating twins, and biological samples have been collected as part of specific sub-studies. The FTCS including the above two cohorts has been described previously (Kaprio (2019), Kaidesoja (2019).**

**The FTCS was established with approval from the National Board of Health and further data collection received ethical approval from the Ethical Committee of the Department of Public Health, University of Helsinki and Ethics Committee of the Hospital District of Helsinki and Uusimaa (#270/13/03/01/2008).**

*Cohort-specific Acknowledgments*

**The Finnish Twin Cohort was supported by a grant (No. 312073) from the Academy of Finland Centre of Excellence in Complex Disease Genetics. Collection of the samples was also funded by: Academy of Finland (#335443, 314383, 272376 and 266286), Sigrid Jusélius Foundation, Finnish Medical Foundation, Finnish Diabetes Research Foundation, Novo Nordisk Foundation (#NNF20OC0060547, NNF17OC0027232, NNF10OC1013354) and Government Research Funds to Helsinki University Hospital.**

*Determination of Type 2 Diabetic Status*

**An individual was categorised as T2D if either there were two or more indicators of T2D (HbA1c** ≥**48, fasting glucose** ≥**7.0, or 2 h glucose in an oral glucose tolerance test** ≥**11.1).**

**The Netherlands Twin Register**

**The Netherlands Twin Register (NTR) consists of around 120,000 twins and approximately as many of their relatives. Twin recruitment began in the 1980s and the majority of twin families have continued to participate in longitudinal surveys with subsets taking part in the biological sample collections. This cohort has been described previously in detail (Ligthart et al, 2019), with more detail on the biobank projects, in which the samples were collected, in Willemsen et al. (2010) and Sirota et al. 2015.**

**The study protocol was approved by Central Ethics Committee on Research involving Human Subjects of the VU University Medical, Center, Amsterdam, an Institutional Review Board certified by the US Office of Human Research Protections (IRB number IRB-2991 under Federal-wide Assurance-3703; IRB/institute codes, NTR 03-180).**

*Cohort-specific Acknowledgments*

**We acknowledge funding from the Netherlands Organization for Scientific Research (NWO) and The Netherlands Organisation for Health Research and Development (ZonMW) grants 904-61-090, 985-10-002, 912-10-020, 904-61-193,480-04-004, 463-06-001, 451-04-034, 400-05-717, Addiction-31160008, 016-115-035, 481-08-011, 056-32-010, Middelgroot-911-09-032, OCW_NWO Gravity program-024.001.003, NWO-Groot 480-15-001/674, Biobanking and Biomolecular Resources Research Infrastructure (BBMRI-NL, 184.021.007 and 184.033.111). The Spinozapremie (NWO-56-464-14192), KNAW Academy Professor Award (PAH/6635) and University Research Fellow grant (URF) to DIB. Amsterdam Public Health and Amsterdam Reproduction and Development research institutes; the European Science Foundation (ESF, EU/QLRT-2001-01254), the European Community’s Seventh Framework Program (FP7-HEALTH-F4-2007-2013, grant 01413: ENGAGE and grant 602768: ACTION); the European Research Council (ERC Advanced 230374), Rutgers University Cell and DNA Repository (NIMH U24 MH068457-06), the National Institutes of Health (NIH, R01D0042157-01A1, R01MH58799-03, MH081802, DA018673, R01 DK092127-04, Grand Opportunity grants 1RC2 MH089951 and 1RC2 MH089995) and the Avera Institute for Human Genetics, Sioux Falls, South Dakota (USA).**

*Determination of Type 2 Diabetic Status*

**An individual was categorised as T2D if the person reported to use T2D medication or to have T2D diabetes in combination with fasting blood glucose levels greater than 7 mmol/L or** hba1c ≥ 6.5, or if the person reported to both TD2 disease and T2D medication and hba1c ≥ 6.

**Murcia Twin Registry**

The Murcia Twin Registry (MTR) was created by a collaboration between the University of Murcia and the regional Health Authority in 2006. At outset the focus was on women’s health but the cohort has been extended to include male twins. There are 3,545 participants from the Spanish region of Murcia in the registry. They were born between 1940 and 1976 and since 2006 there have been five rounds of data collection including surveys and biological samples. The cohort and data collected have been previously described (Ordoñana et al, 2019).

Since its inception, the MTR has been administered and managed by the Area of Psychobiology of the University of Murcia and its procedures have been approved by the Murcia University Ethical Committee.

*Cohort-specific Acknowledgments*

The MTR is being funded by Seneca Foundation — Regional Agency for Science and Technology, Murcia, Spain (03082/PHCS/05, Ref.100/2005; 08633/PHCS/08, Ref.359/2008; 15302/PHCS/10, Ref.617/2012; and 19479/PI/14, Ref.1213/2016) and the Spanish Ministry of Science and Innovation (PSI2009–11560, Ref.417/2019; PSI2014-56680-R, Ref.1032/2015; and RTI2018-095185-B-I00, Ref.2098/2018).  co-funded by European Regional Development Fund (FEDER). The funder had no role in the study design, data collection, data analyses, writing, or the decision to submit this manuscript for publication. Approval from the Ethics Committee was obtained also specifically for this project (Ref.1031/2015).

*Determination of Type 2 Diabetic Status*

**An individual was categorised as T2D if the person reported having been diagnosed by a medical doctor or use T2D medication. This information was confirmed by medical records from a regional health register regarding fasting blood glucose levels, kind of medication and medical follow-up by a primary care physician.**

**Danish Twin Registry**

The Danish Twin Registry (DTR) is the oldest national twin registry originally recruiting twins born between 1870 and 1910 and now including twins born up until 2009. There are 140 birth cohorts in total consisting of around 175,000 twins. The data consists of surveys conducted over time and biological samples collected since 1997. The cohort and data collected have been previously described (Pedersen et al, 2019).

The study has been approved by The Regional Scientific Ethical Committees for Southern Denmark (S-20150003, S-20090065) and The Danish Data Protection Agency (1999-1200-441, 2009-41-2990)

*Cohort-specific Acknowledgments*

We want to thank the following for their considerable financial contribution to the foundation and the continued support of the Danish Twin Registry: the Danish Research Council, University of Southern Denmark, the Danish National Research Foundation, the Danish Cancer Society, the Danish Diabetic Association, the Danish Heart Foundation, the Novo Nordisk Foundation, United States National Institute of Aging (P01AG08761), United States National Cancer Institute, the National Program for Research Infrastructure 2007 from the Danish Agency for Science Technology and Innovation, and Interreg 4a Southern

Denmark-Schleswig-KERN funded by the European Regional Development

Fund (grant number 57-1.3-10).

*Determination of Type 2 Diabetic Status*

Type 2 diabetes was defined as self-reported of having been diagnosed with diabetes, FPG ≥ 7.0 mmol/l, HbA1c ≥ 48 mmol/mol (6.5%) or self-reported use of antidiabetic medicine**.**

**The Swedish Twin Registry**

Between 2004 and 2008, twin-pairs who had participated in a previous national telephone interview were invited to respond to a paper health questionnaire about common diseases and to provide a blood sample and have their blood pressure, height and weight measured at their local healthcare facility. Out of 22,000 twins invited, close to 14,600 (65%) responded to the questionnaire. Questions about diabetes included type of diabetes and diabetes medication and whether (and when) or not a medical doctor had established the diagnosis. Additional medical information is obtained directly from national health registers. The cohort and data collected have been previously described (Zagai et al, 2019).

Informed consent was obtained for this study and the study was approved by the Regional Ethics Board of Stockholm (2007-644-31-2).

*Cohort-specific Acknowledgments*

The Swedish Twin Registry is managed by Karolinska Institutet and receives funding through the Swedish Research Council as a national research infrastructure under the grant 2021-00180.

*Determination of Type 2 Diabetic Status*

**The selection of same-sexed twin from the Swedish Twin Registry was based on T2D discordance. A twin-pair was categorised as T2D discordant if one of the twins had diabetes diagnosis in the National patient register or had self-reported type 2 diabetes either diagnosed by a physician or combined with records of prescribed diabetes medication. The healthy co-twin had to be free from all these criteria. In 22 out of 23 discordant pairs the affected twin had a diagnosis in the National patient register.**

## DNA methylation profiling

The protocol for using the DNA methylation panel has also been described previously (Allum et al, 2015). Following preparation, bisulfite conversion and amplification of the MCC-seq library the targeted DNA fragments are captured based on SeqCap Epi Enrichment System protocol (Roche NimbleGen), amplified again and sequenced. Briefly, genomic DNA (gDNA) was isolated, amplified, set-up and normalized at 1000ng spiked with 0.1% (w/w) unmethylated λ DNA (Promega) using Beckman Coulter's Biomek. After DNA extraction, whole- genome sequencing libraries were fragmented into 300–400 bp peak sizes using the covaries focused-ultrasonicator E210. Fragment size was controlled on a Bioanalyzer DNA 1000 Chip (agilent) and the library preparation was applied on Sciclone (PerkinElmer). Fragments were divided to be multiplexed and incubated at 47 °C for 72 h. End repair of the generated dsDNA with 3ʹ or 5ʹ overhangs, adenylation of 3ʹ ends, adaptor ligation, and clean-up steps were carried out as per PerkinElmer’ recommendations. The cleaned-up ligation product was then analyzed on a Bioanalyzer High Sensitivity DNA Chip (Agilent) and quantified by PicoGreen (Life Technologies). Samples were then bisulfite converted using the Epitect Fast DNA Bisulfite Kit according to the manufacturer’s protocol. Bisulfite-converted DNA was quantified using OliGreen (Life Technologies) and, based on quantity, amplified by 9–12 cycles of PCR using the Kapa Hifi Uracil + DNA polymerase (KAPA Biosystems) according to the manufacturer’s protocol. The amplified libraries were purified using Ampure Beads distribution of the captured library were validated on Bioanalyzer High Sensitivity DNA Chips and quantified by PicoGreen. To make a capture, 10 to 12 libraries were pooled together and then sequenced on the Illumina HiSeq2000/2500 system using 100-bp paired-end sequencing or on the Illumina HiSeq4000 system using 150-bp paired-end sequencing.

In-house generated methylome libraries were aligned using after converting all the reads in bisulfite mode to the bisulfited-converted human hg19/GRCh37 genome reference and Lambda and pUC19 sequences, which were added as bisulfite conversion control set. Both reads in a pair were trimmed of any low-quality sequence at their 3′ ends (with Phred scale score ≥30). Post-process read mappings were made as previously described (Johnson et al, Currr Protoc Mol Bio, 2012), including clipping 3′ ends of overlapping read pairs in both forward and reverse strand mappings, filtering duplicate, low-mapping quality reads (MQ<20), and read pairs not mapped at the expected distance based on the library insert size, as well as reads with more than 2% mismatches. Methylation calls of individual CpGs were extracted using Bis-SNP. CpGs located within ENCODE DAC blacklisted regions or Duke excluded regions were discarded. For each sample, CpG sites with the number of total reads less than 5 were discarded. CpG sites with more 2 reads in both strands and more than 5 with the number of total reads were also discarded when the difference of methylation percent between two strands is more than 20% in absolute value (called hemi-methylated and high coverage CpG site). Thus, we have only fully methylated and high coverage CpG sites that were further studied. We combined all samples and then removed all CpG sites that overlap the list of UK10k’s variants that have a MAF above 5. We further excluded sites where less than 80% of participants had methylation readings. Overall, this included 2,048,698 sites.

**References**

[Effect of genome and environment on metabolic and inflammatory profiles.](https://pubmed.ncbi.nlm.nih.gov/25853885/)

Sirota M, Willemsen G, Sundar P, Pitts SJ, Potluri S, Prifti E, Kennedy S, Ehrlich SD, Neuteboom J, Kluft C, Malone KE, Cox DR, de Geus EJ, Boomsma DI.PLoS One. 2015 Apr 8;10(4):e0120898. doi: 10.1371/journal.pone.0120898.

[The Netherlands Twin Register biobank: a resource for genetic epidemiological studies.](https://pubmed.ncbi.nlm.nih.gov/20477721/)

Willemsen G, de Geus EJ, Bartels M, van Beijsterveldt CE, Brooks AI, Estourgie-van Burk GF, Fugman DA, Hoekstra C, Hottenga JJ, Kluft K, Meijer P, Montgomery GW, Rizzu P, Sondervan D, Smit AB, Spijker S, Suchiman HE, Tischfield JA, Lehner T, Slagboom PE, Boomsma DI.Twin Res Hum Genet. 2010 Jun;13(3):231-45. doi: 10.1375/twin.13.3.231

[The Netherlands Twin Register: Longitudinal Research Based on Twin and Twin-Family Designs.](https://pubmed.ncbi.nlm.nih.gov/31666148/)

Ligthart L, van Beijsterveldt CEM, Kevenaar ST, de Zeeuw E, van Bergen E, Bruins S, Pool R, Helmer Q, van Dongen J, Hottenga JJ, Van't Ent D, Dolan CV, Davies GE, Ehli EA, Bartels M, Willemsen G, de Geus EJC, Boomsma DI.Twin Res Hum Genet. 2019 Dec;22(6):623-636. doi: 10.1017/thg.2019.93.

Sachdev, P.S., et al. A comprehensive neuropsychiatric study of elderly twins: the Older Australian Twins Study. Twin Res Hum Genet 12, 573-582 (2009).

Zagai U, Lichtenstein P, Pedersen NL, Magnusson PKE. The Swedish Twin Registry: Content and Management as a Research Infrastructure. Twin Res Hum Genet. 2019 Dec;22(6):672-680. doi: 10.1017/thg.2019.99. Epub 2019 Nov 21. PMID: 31747977

Kaidesoja M, Aaltonen S, Bogl LH, Heikkilä K, Kaartinen S, Kujala UM, Kärkkäinen U, Masip G, Mustelin L, Palviainen T, Pietiläinen KH, Rottensteiner M, Sipilä PN, Rose RJ, Keski-Rahkonen A, Kaprio J. FinnTwin16: A Longitudinal Study from Age 16 of a Population-Based Finnish Twin Cohort. Twin Res Hum Genet. 2019 ;22(6):530-539. doi: 10.1017/thg.2019.106. PMID: 31796134.

Kaprio J, Bollepalli S, Buchwald J, Iso-Markku P, Korhonen T, Kovanen V, Kujala U, Laakkonen EK, Latvala A, Leskinen T, Lindgren N, Ollikainen M, Piirtola M, Rantanen T, Rinne J, Rose RJ, Sillanpää E, Silventoinen K, Sipilä S, Viljanen A, Vuoksimaa E, Waller K. The Older Finnish Twin Cohort - 45 Years of Follow-up. Twin Res Hum Genet. 2019;22(4):240-254. doi: 10.1017/thg.2019.54.. PMID: 31462340.
